# Gut Mucosal Microbiome Is Perturbed in Rheumatoid Arthritis Mice and Partly Restored after TDAG8 Deficiency or Suppression by Salicylanilide Derivative

**DOI:** 10.3390/ijms23073527

**Published:** 2022-03-24

**Authors:** Ngoc Tuan Nguyen, Wei-Hsin Sun, Tzu-Hsuan Chen, Po-Chun Tsai, Chih-Chen Chen, Shir-Ly Huang

**Affiliations:** 1 Faculty of Applied Sciences, Ton Duc Thang University, Ho Chi Minh City 700000, Vietnam; nguyenngoctuan@tdtu.edu.vn; 2 Department of Life Sciences and Institute of Genome Sciences, National Yang Ming Chiao Tung University, Taipei 112, Taiwan; weihsin@nycu.edu.tw (W.-H.S.); gina931582@gmail.com (T.-H.C.); 3 Institute of Microbiology and Immunology, National Yang Ming Chiao Tung University, Taipei 112, Taiwan; hijikatasun.ls05@nycu.edu.tw (P.-C.T.); chihchen29@nycu.edu.tw (C.-C.C.)

**Keywords:** rheumatoid arthritis, pain, T-cell death-associated gene 8, microbiome, microbial diversity

## Abstract

Rheumatoid arthritis (RA), an autoimmune disease, is characterized by chronic joint inflammation and pain. We previously found that the deletion of T-cell death-associated gene 8 (TDAG8) significantly reduces disease severity and pain in RA mice. Whether it is by modulating gut microbiota remains unclear. In this study, 64 intestinal samples of feces, cecal content, and cecal mucus from the complete Freund’s adjuvant-induced arthritis mouse models were compared. The *α*- and *β*-diversity indices of the microbiome were significantly lower in RA mice. Cecal mucus showed a higher ratio of *Firmicutes* to *Bacteroidetes* in RA than healthy mice, suggesting the ratio could serve as an RA indicator. Four core genera, *Eubacterium_Ventriosum*, *Alloprevotella*, *Rikenella*, and *Treponema*, were reduced in content in both feces and mucus RA samples, and could serve microbial markers representing RA progression. TDAG8 deficiency decreased the abundance of proinflammation-related *Eubacterium_Xylanophilum*, *Clostridia*, *Ruminococcus*, *Paraprevotella*, and *Rikenellaceae*, which reduced local mucosal inflammation to relieve RA disease severity and pain. The pharmacological block of the TDAG8 function by a salicylanilide derivative partly restored the RA microbiome to a healthy composition. These findings provide a further understanding of specific bacteria interactions with host gut mucus in the RA model. The modulation by TDAG8 on particular bacteria can facilitate microbiota-based therapy.

## 1. Introduction

Rheumatoid arthritis (RA) is a common, autoimmune, inflammatory, and chronic disease that affects nearly 1% of the adult population worldwide [1,2]. RA is characterized by its severely progressive disability, systemic complications, early death, and health expenditure terms. The pathogenesis of RA is complex and leads to the destruction of both cartilaginous and bony elements of the joint. The dysregulated inflammatory processes in the synovium of the joint are often accompanied by ongoing pain and increased pain during movement. The etiology of RA is ambiguous; the initiation of RA seems to result from both genetic and environmental causes [1,2].

Various risk factors have been indicated as potential causes for RA, and microorganisms have recently been of interest as a risk factor. The overrepresentation of some microorganisms in the intestines could be related to RA morbidity. In fact, fluctuations in bacterial content might lead to altered levels of metabolites that promote joint inflammation [3,4,5]. Vaahtovuo et al. used fecal samples to investigate bacterial composition based on DNA staining, flow cytometry, and 16S rDNA hybridization in RA patients. RA patients had a significantly lower content of bifidobacteria and bacteria of the *Bacteroides-Porphyromonas-Prevotella* group, *Eubacterium rectale*-*Clostridium coccoides* group, and *Bacteroides fragilis* subgroup [6]. Scher et al. used the V1–V2 variable region of bacterial 16S rDNA gene amplification and found the presence of *Prevotella* as highly correlated with disease in patients with new-onset untreated RA [7]. Zhang et al. used metagenomic shotgun sequencing to study the microbiome of fecal, dental, and salivary samples from an RA cohort and healthy controls. The genus *Haemophilus* was depleted in RA patients, whereas that of *Lactobacillus* was increased in individuals with RA in all three samples. A cluster containing the genera *Klebsiella*, *Bifidobacterium*, *Sutterella*, and *Megamonas* was enriched in healthy controls. In contrast, a large group including the genera *Gordonibacter*, *Clostridium*, *Lachnospiraceae*, and *Eggerthella* was enriched in RA patients [8]. Jeong et al. reported the overgrowth of the genus *Collinsella* in healthy individuals [9]. Sun et al. found that RA patients showed an increase in the content of 8 bacterial genera (*Bacteroides*, *Escherichia-Shigella*, the *Eubacterium xylanophium* group, *Flavonifractor*, *Oscillospira*, *Parasutterella*, *Sellimonas*, and *Tyzzerella*) and a decrease in 18 genera *Akkermansia*, *Alloprevotella*, *Coprococcus* 1, *Coriobacteriaceae* UCG-002, *Citrobacter*, *Clostridium* sensu stricto-3, *Desulfovibrio*, *Enterobacter*, *Enterococcus*, *Helicobacter*, *Klebsiella*, *Lactobacillus*, *Odoribacter*, *Rikenellaceae* RC9, *Rikenella*, *Ruminococcaceae* UCG-014, *Rhodococcus*, and *Staphylococcus* [10].

To understand the mechanisms of the disease and evaluate therapeutic targets, several arthritis mouse models have been established [11,12,13,14]. We adopted and modified Gauldie’s method to establish an arthritis model that could reproduce some of the possible mechanisms at play in RA [15]. Several proton-sensing receptors, including transient receptor potential/vanilloid receptor subtype 1 (TRPV1), acid-sensing ion channel 3 (ASIC3), and proton-sensing G-protein-coupled receptors, were found to be associated with arthritis or arthritis-associated pain [16,17,18]. We previously demonstrated that ASIC3, TRPV1, and T-cell death-associated gene 8 (TDAG8) modulate RA disease progression and RA-associated pain [15]. TDAG8 gene deletion reduces RA disease severity and relieves RA-associated pain through the regulation of satellite glial cells and proinflammatory macrophages [19]. Small molecule compounds (such as CCL-2d, LCC-09, and NSC745885) inhibit TDAG8 gene expression and function, also relieving RA-associated pain [19,20]. Genetic variants in the TDAG8 locus are associated with spondyloarthritis [21], TDAG8 is highly expressed in Th17 cells [22], and TDAG8 deletion reduces Th17 cell number and IL 17 secretion [23]. RA has been proposed to start at the mucus site with inflammation and autoimmunity, which responds to microbes or microbial factors. However, the characteristics of the microbiome in mucus and its role modulating RA is limited. Furthermore, the role of the cecal content and cecal mucus microbiome in RA is still unknown. In this study, we used a previously established RA mouse model induced by complete Freund’s adjuvant [15] to collect 64 samples from three sites, including feces, cecal content, and cecal mucus. We studied the microbial composition and the association of the microbiome in feces, cecal content, and cecal mucus. We also analyzed microbial composition in TDAG8-deficient and TDAG8 inhibitor (CCL-2d)-treated RA mice for relieving RA disease severity and pain. TDAG8 gene deletion or inhibition restored the altered microbial composition to a healthy condition, so TDAG8 may regulate gut microbiota to modulate RA disease progression and pain. Accordingly, these findings provided us with a more fundamental understanding of microbial composition in RA and TDAG8 modulation in RA through microbiota. It could facilitate the development of novel therapies in RA and RA pain by both novel small molecules and bacteria.

## 2. Results

### 2.1. Differences in Microbiomes of RA Mice

To examine the association between RA and changes in microbial profiles, total DNA from samples from three locations, feces, cecal content, and cecal mucus, was extracted and sequenced. The 3,461,121 reads were grouped into 1110 OTUs, with a mean of 59,674 reads across the 58 samples. Good’s coverage was high, with an average of 0.999 across all samples. Reads distributed by samples are described in Table 1.

**Figure 1:**
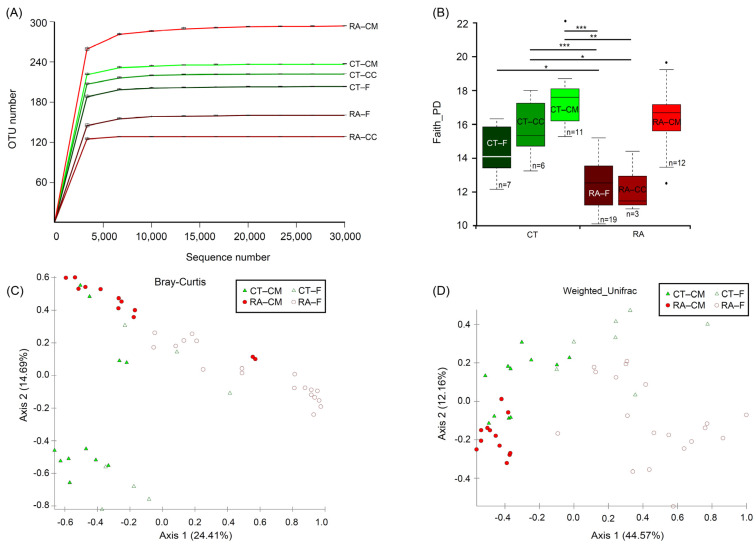
Alpha- and *β*-diversity analysis between RA and healthy ICR mice. (**A**) Rarefaction analysis of 16S rDNA data from RA mice and healthy controls. Each line represents fecal, cecal content and cecal mucus samples. Samples were rarified at an even depth of 31,794 sequences per sample for further analysis. Operational taxonomical units (OTUs) in this analysis were defined at 99% similarity. (**B**) Faith’s Phylogenetic Diversity comparisons were calculated by Kruskal–Wallis test. Data are median (horizontal line), interquartile range (box edges) and range (whiskers). * *p* < 0.05, ** *p* < 0.01, *** *p* < 0.001. (**C**) Principal coordinate analysis plot constructed by using the Bray–Curtis distance matrix. (**D**) Principal coordinate analysis plot constructed by using the weighted-UniFrac distance matrix. CT, healthy controls; RA, rheumatoid arthritis; CM, cecal mucus; CC, cecal content; F, fecal. All mice were from ICR background.

The complete and resampled datasets were used to calculate the Bray–Curtis dissimilarity, and then the Mantel test was used to compare both datasets. The results showed a significant correlation (correlation coefficient 0.9802, *p* = 0.001) and indicated a difference in the number of sequences per sample, causing no effects in the analysis. In addition, the α-diversity revealed no major differences for both matrices (Table 1). Analyses of microbial communities revealed differences in richness (observed OTUs) between RA and healthy mice (Figure 1A). To standardize the microbiome measures, a minimum of 31,794 sequences was used per sample. Rarefaction curves that reached the plateau phase indicated that the sequencing depth was sufficient for an analysis (Figure 1A). Faith’s Phylogenetic Diversity was statistically significant in the microbiome from RA mice through healthy controls (*p* < 0.05) for both feces and cecal content; however, the cecal mucus content did not differ between healthy controls and RA mice (Figure 1B). The significant variations in the microbial diversity at different sites from the same mouse group were analyzed. The bacterial diversity in the cecal mucus resulted in significant differences (*p* < 0.05) in the microbiome of feces or cecal content in RA mice. Diversity did not differ between feces and the cecal content for mouse groups, either the control or RA model.

To investigate both community evenness and richness, the Shannon diversity index revealed a similar decreasing trend in phylogenetic differences in different groups, but only feces revealed a significant difference (*p* = 0.04, Kruskal–Wallis) between control and RA mice. The microbial community was markedly less diverse in RA than control mice. Therefore, the development of RA might be related to a decline in the α-diversity of the microbiome. The data from the feces samples agreed with data from the cecal content samples. Therefore, the cecal content samples were ignored and a further analysis focused on the data from the fecal and cecal mucus samples. Healthy controls and RA mice significantly differed in bacterial community in feces (PERMANOVA, *p* < 0.05) by using the Bray–Curtis distance-based microbiome structure analysis, separating along principal coordinate dimension 1 (PCoA1) and explaining approximately 24.4% of the total variations in data. They also differed in bacterial community in cecal mucus (PERMANOVA, *p* < 0.05) (Figure 1C). PERMDISP indicated that dispersion did not contribute to significance (Table S1). The principal coordinate analysis of the weighted UniFrac distance showed that the RA treatment compared to healthy mice resulted in significant differences in *β*-diversity in both feces and mucus (PERMANOVA, *p* < 0.05), separating along principal coordinate dimension 1 (PCoA1) and explaining approximately 44.5% of the total variations in data. They also differed in bacterial community in cecal mucus (PERMANOVA, *p* < 0.05) (Figure 1D). Similar results were obtained from the microbiome structure analysis when using the unweighted UniFrac and Jaccard distance (Figure S1). Considering these results, we found evidence for RA-associated differences in both *α*- and *β*-diversity in bacterial community between the fecal and cecal mucus samples.

### 2.2. Core Microbiome

**Figure 2:**
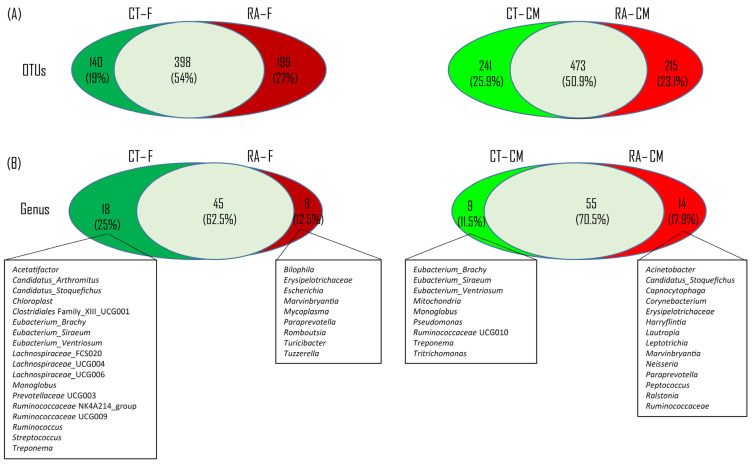
Venn diagram of bacterial content. (**A**) Common and unique OTUs between healthy control and RA mouse samples in each intestinal location. (**B**) Common and unique genera across two intestinal sites.

To test the presence of an identifiable common core bacterial community defined as the shared members among the microbiome and common genera, we used a Venn diagram. We identified 538 and 714 OTUs in feces and cecal mucus, respectively, from healthy mice, and 597 and 688 OTUs in feces and cecal mucus from RA mice (Figure 2A). In total, 398 OTUs were shared between healthy and RA groups, occupying 54% of all OTUs (737 OTUs) in feces, whereas 473 OTUs were shared between the healthy control and RA mice, representing 50.9% of all OTUs (929 OTUs) in cecal mucus. At the genus level, we identified 63 and 64 genera in feces and cecal mucus from healthy mice, and 54 and 69 genera in feces and cecal mucus from RA mice (Figure 2B). In total, 45 and 55 genera were shared between healthy and RA groups, representing 62.5% and 70.5% of all genera in feces and cecal mucus, respectively.

In fecal samples, we observed 18 unique genera in healthy controls, including *Acetatifactor*, *Candidatus_Arthromitus*, *Candidatus_Stoquefichus*, *Chloroplast*, *Clostridiales_UCG001*, *Eubacterium_Brachy*, *Eubacterium_Siraeum*, *Eubacterium_Ventriosum*, *Lachnospiraceae*_FCS020, *Lachnospiraceae*_UCG004, *Lachnospiraceae*_UCG006, *Monoglobus*, *Prevotellaceae* UCG003, *Ruminococcaceae* NK4A214, *Ruminococcaceae* UCG009, *Ruminococcus*, *Streptococcus*, and *Treponem*, and 9 unique genera in RA mice, including *Bilophila*, *Erysipelotrichaceae*, *Escherichia*, *Marvinbryantia*, *Mycoplasma*, *Paraprevotella*, *Romboutsia*, *Turicibacter*, and *Tuzzerella* (Figure 2B). In cecal mucus samples, 9 unique genera in healthy controls were observed, including *Eubacterium_Brachy*, *Eubacterium_Siraeum*, *Eubacterium_Ventriosum*, *Mitochondria*, *Monoglobus*, *Pseudomonas*, *Ruminococcaceae* UCG010, *Treponema*, and *Tritrichomonas*, and 14 unique genera in RA mice, including *Acinetobacter*, *Candidatus_Stoquefichus*, *Capnocytophaga*, *Corynebacterium*, *Erysipelotrichaceae*, *Harryflintia*, *Lautropia*, *Leptotrichia*, *Marvinbryantia*, *Neisseria*, *Paraprevotella*, *Peptococcus*, *Ralstonia*, and *Ruminococcaceae* (Figure 2B).

All things considered, five and three common unique genera were found in both feces and cecal mucus samples, respectively, from healthy controls and RA mice. The five common unique genera in both the feces and cecal mucus from healthy controls belonged to *Eubacterium_Brachy*, *Eubacterium_Siraeum*, *Eubacterium_Ventriosum*, *Monoglobus*, and *Treponema*. *Erysipelotrichaceae*, *Marvinbryantia*, and *Paraprevotella* were not observed in healthy controls as compared with their abundance in RA mice (Figure 2B).

### 2.3. Featured Microbial Taxa by Using LEfSe

**Figure 3:**
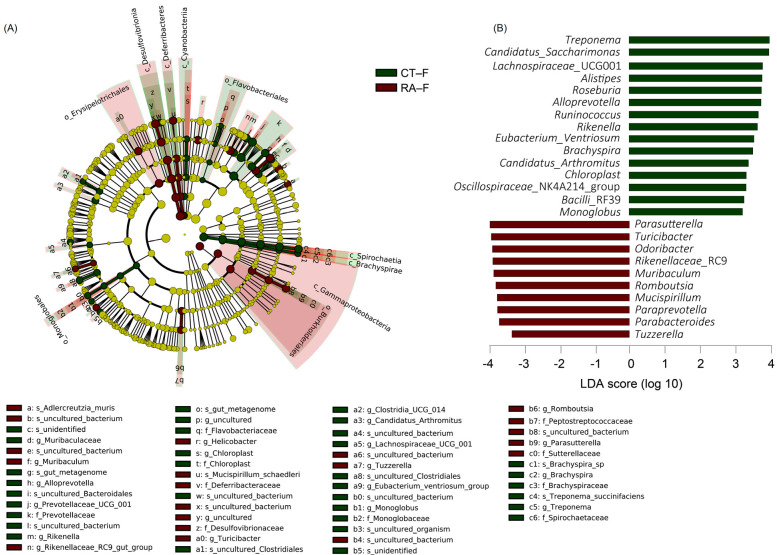
Linear discriminant analysis effect size (LEfSe) analysis of fecal microbiome in healthy control and RA mouse samples. (**A**) Cladogram constructed by LEfSe indicating alternations between healthy control and RA samples. Regions in green indicate taxa enriched in healthy controls and regions in red indicate taxa enriched in RA mice. The bottom of the cladogram shows the differing taxa. (**B**) LDA scores are described in a bar graph.

An LDA (score > 3) effect size-based cladogram showed bacterial species enriched in feces from healthy controls: *Spirochaetes* and *Patescibacteria* phyla; *Spirochaetia*, *Cyanophyceae*, and *Brachyspirae* class; *Spirochaetaceae* and *Brachyspiraceae* families; *Muribaculaceae*, *Alloprevotella*, *Prevotellaceae*_UCG_001, *Rikenella*, *Chloroplast*, *Clostridia*_UCG_014, *Candidatus_Arthromitus*, *Lachnospiraceae*_UCG_001, *Eubacterium_Ventriosum*, *Monoglobus*, *Brachyspira*, and *Treponema* genera (Figure 3A). Bacterial species enriched in feces from RA mice were taxa in the *Proteobacteria* and *Desulfobacterota* phyla; *γ-proteobacteria*, *Desulfovibrionia*, and *Deferribacteres* class; *Deferribacteraceae*, *Desulfovibrionaceae*, *Peptostreptococcaceae*, and *Sutterellaceae* families; *Muribaculum*, *Rikenellaceae*_RC9, *Helicobacter*, *Turicibacter*, *Tuzzerella*, *Romboutsia*, and *Parasutterella* genera. The data were also indicated in an LDA bar graph (Figure 3B): 15 genera enriched in fecal samples from healthy controls included *Treponema*, *Candidatus_Saccharimonas*, *Lachnospiraceae*_UCG001, *Alistipes*, *Roseburia*, *Alloprevotella*, *Runinococcus*, *Rikenella*, *Eubacterium_Ventriosum*, *Brachyspira*, *Candidatus_Arthromitus*, *Chloroplast*, *Oscillospiraceae*_NK4A214, *Bacilli*_RF39, and *Monoglobus*, and 10 genera enriched in feces from RA mice included *Parasutterella*, *Turicibacter*, *Odoribacter*, *Rikenellaceae*_RC9, *Muribaculum*, *Romboutsia*, *Mucispirillum*, *Paraprevotella*, *Parabacteroides*, and *Tuzzerella* (Figure 3B). Similar to core microbiome results in fecal samples, genera including *Candidatus_Arthromitus*, *Chloroplast*, *Monoglobus*, and *Treponema* had different LDA scores in healthy controls, whereas *Turicibacter*, *Romboutsia*, *Paraprevotella*, and *Tuzzerella* had different LDA scores in RA mice.

**Figure 4:**
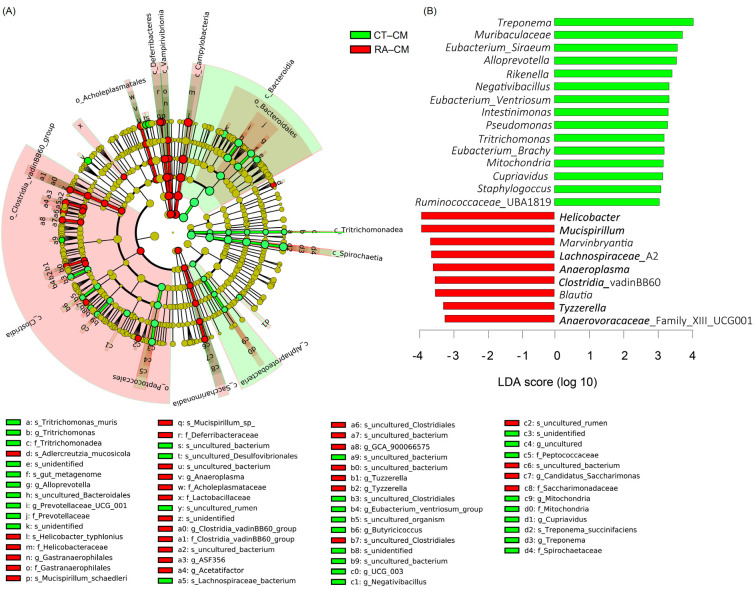
LEfSe analysis of cecal mucosa microbiome in healthy control and RA mouse samples. (**A**) Cladogram constructed by LEfSe indicating alternations between healthy control and RA samples. Regions in green indicate taxa enriched in healthy controls and regions in red indicate taxa enriched in RA mice. The bottom of the cladogram shows the differing taxa. (**B**) LDA scores are described in a bar graph.

For cecal mucus, species enriched in healthy controls were in the *Bacteroidetes* and *Spirochaetes* phyla; *Bacteroidia*, *Spirochaetia*, and *α-proteobacteria* classes; *Prevotellaceae*, *Peptococcaceae*, *Mitochondria*, and *Spirochaetaceae* families; *Alloprovotella*, *Provotellaceae*_UGC_001, *Eubacterium_Ventriosum*, *Butyricicoccus*, *Negativibacillus*, *Mitochondria*, *Cupriavidus*, and *Treponema* genera, whereas species enriched in RA mice were in the *Deferribacteres*, *Cyanobacteria*, and *Campilobacterota* phyla; *Deferribacteres*, *Vampirvibrionia*, *Campylobacteria*, *Clostridia*, and *Saccharimonadia* classes; *Gastranaerophilales*, *Anaeroplasma*, *Clostridia*_vadinBB60, *Acetatifactor*, *Tuzzerella*, *Tyzzerella*, and *Candidatus_Saccharimonas* genera (Figure 4A). These results were also illustrated in an LDA bar graph (Figure 4B). Fifteen genera enriched in cecal mucus samples from healthy controls included *Treponema*, *Muribaculaceae*, *Eubacterium_Siraeum*, Alloprevotella, Rikenella, Negativibacillus, *Eubacterium_Ventriosum*, *Intestinimonas*, *Pseudomonas*, *Tritrichomonas*, *Eubacterium_Brachy*, *Mitochondria*, *Cupriavidus*, *Staphylococcus*, and *Ruminococcaceae*_UBA1819, whereas nine genera enriched in cecal mucus samples from RA mice included *Helicobacter*, *Mucispirillum*, *Marvinbryantia*, *Lachnospiraceae*_A2, *Anaeroplasma*, *Clostridia*, *Blautia*, *Tyzzerella*, and *Anaerovoracaceae*_ UCG001 (Figure 4B). Similar to core microbiome results in cecal mucus samples, the genera including *Mitochondria*, *Pseudomonas*, *Treponema*, and *Tritrichomonas* revealed different LDA scores in healthy controls, and *Marvinbryantia* yielded different LDA scores in RA mice.

### 2.4. Featured Microbial Taxa Using Wilcoxon Rank-Sum Tests

To further examine the variation in the relative abundance of different microbial taxa between the healthy control and RA groups, we used comparative analyses at all taxonomic levels for the mean relative abundance of two groups. The bacterial composition of each group at both the phylum and genus levels was analyzed by Wilcoxon rank-sum tests to identify taxa differing in abundance at phylum levels (Table 2). A total of 16 bacterial phyla was obtained in all samples; the phyla *Firmicutes* and *Bacteroidetes* were the most abundant, representing >80% of the gut microbiome. The present data showed a significantly increased abundance of *Spirochaetes* and *Patescibacteria*, and a decreased abundance of *Proteobacteria* and *Desulfobacterota* in fecal samples from RA mice (Table 2).

**Figure 5:**
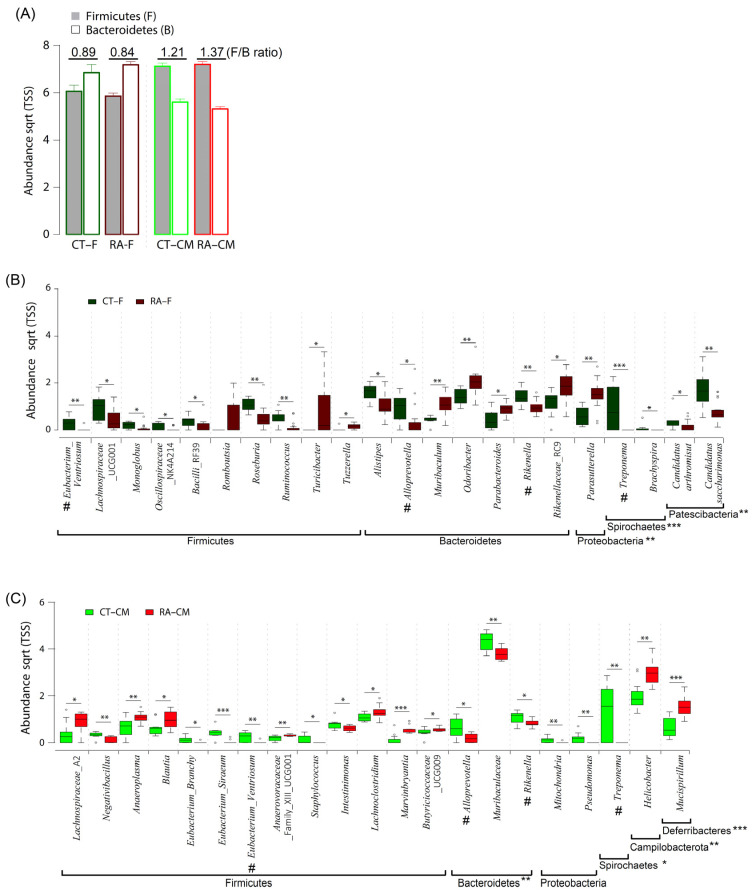
Composition of gut microbiome in healthy controls and RA mice. (**A**) The ratio of *Firmicutes* to *Bacteroidetes* in cecal mucus samples. (**B**) Significantly abundant genera in fecal samples. (**C**) Significantly abundant genera in cecal samples. Data are median (horizontal line), interquartile range (box edges) and range (whiskers). * *p* < 0.05; ** *p* < 0.01; *** *p* < 0.001, Wilcoxon rank-sum test. The same genera between fecal samples and cecal mucus samples are indicated by #.

The ratio of *Firmicutes/Bacteroidetes* (*F/B*) is considered an important marker of the gut microbiome state. In this study, RA mice had a lower *F/B* ratio in fecal samples as compared to healthy controls (Figure 5A). At the genus level, we identified 21 differentially abundant genera in fecal samples, with 14 genera enriched in healthy controls (*Eubacterium_Ventriosum*, *Lachnospiraceae*_UCG001, *Monoglobus*, *Oscillospiraceae*_NK4A214 group; *Bacilli*_RF39, *Roseburia*, *Ruminococcus*, *Alistipes*, *Alloprevotella*, *Rikenella*, *Treponema*, *Brachyspira*, *Candidatus_Saccharimonas*, and *Candidatus_Arthromis*) and 7 genera (*Turicibacter*, *Tuzzerella*, *Muribaculum*, *Odoribacter*, *Parabacteroides*, *Rikenellaceae*_RC9, and *Parasutterella*) enriched in RA (Figure 5B and Table 3). Within the phylum *Firmicutes*, the genera *Eubacterium_Ventriosum*, *Roseburia*, and *Ruminococcus* were more abundant (*p* ≤ 0.01) in healthy controls than in RA mice. Within the phylum *Bacteroidetes*, *Proteobacteria*, *Patescibacteria*, and *Spirochaetes*, the genera *Rikenella*, *Candidatus_Saccharimonas*, and *Treponema* were more abundant (*p* ≤ 0.01) in healthy controls than in RA mice.

For cecal mucus samples, three differentially abundant taxa enriched in healthy controls included *Parabasalia*, *Bacteroidetes*, and *Spirochaetes*, whereas *Campilobacterota* and *Deferribacteres* were enriched in RA mice (Table 2). The statistical analysis revealed significant differences in the relative abundance of *Bacteroidetes*, with an average of a 27.9% and 33.8% abundance for RA samples and healthy controls, respectively. The *F/B* ratio was higher in cecal mucus samples in RA mice than healthy controls (Figure 5A). At the genus level, 20 differentially abundant genera included 12 genera enriched in healthy controls (*Negativibacillus*, *Eubacterium_Brachy*, *Eubacterium_Siraeum*, *Eubacterium_Ventriosum*, *Staphylococcus*, *Intestinimonas*, *Alloprevotella*, *Muribaculaceae*, *Rikenella*, *Mitochondria*, *Pseudomonas*, and *Treponema*) and 8 genera enriched in RA mice (*Lachnospiraceae*_A2, *Anaeroplasma*, *Blautia*, *Lachnoclostridium*, *Marvinbryantia*, *Butyricicoccaceae*_UCG009, *Helicobacter*, and *Mucispirillum*). Within the phylum *Firmicutes*, the bacterial genera *Eubacterium_Siraeum*, *Eubacterium_Ventriosum*, and *Negativibacillus* were more abundant (*p* ≤ 0.01) in healthy controls than in RA mice. Within the phylum *Proteobacteria*, the genera *Mitochondria* and *Pseudomonas* were more abundant (*p* ≤ 0.01) in healthy controls than RA mice. However, the *Treponema* and *Muribaculaceae* genera were more abundant (*p* ≤ 0.01) in healthy controls than RA mice (Figure 5C and Table 3).

### 2.5. Restoration of Microbiome in Cecal Mucosa in TDAG8^−/−^ and CCL-2d-Treated Mice

**Figure 6:**
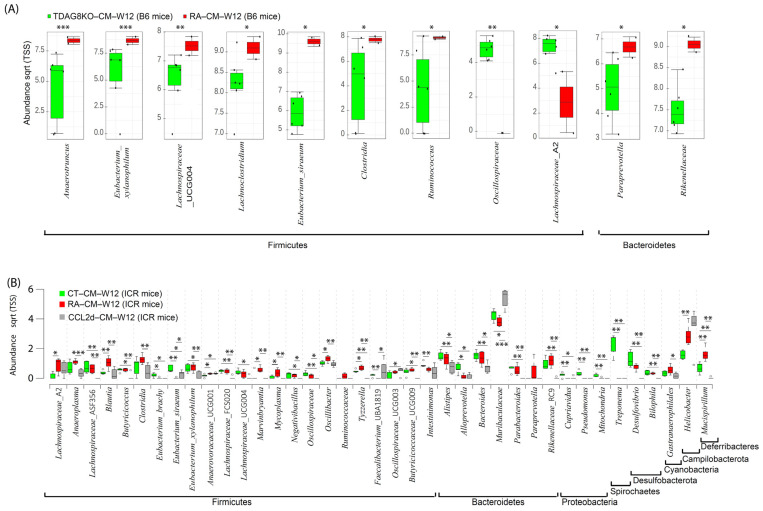
Composition of the cecal mucosa microbiome in healthy control, RA, TDAG8-deficient, and CCL-2d-treated mice at week 12. (**A**) Significantly abundant genera in RA and TDAG8-deficient B6 mice. (**B**) Significantly abundant genera in healthy control, RA, and CCL-2d treated ICR mice. Data are median (horizontal line), interquartile range (box edges) and range (whiskers). * *p* < 0.05; ** *p* < 0.01; *** *p* < 0.001, Wilcoxon rank-sum test.

To obtain the new insight into the pathology of RA, the Wilcoxon rank-sum test was used to investigate taxa differing in abundance between healthy and RA mouse groups at week 12 after the first CFA injection in TDAG8^−/−^ and CCL-2d-treated mice (Figure 6). Using B6 mice treated with CFA, we identified 11 differentially abundant taxa in cecal mucus samples between TDAG8^+/+^ mice and TDAG8^−/−^ mice (Figure 6A). Within the phylum *Firmicutes*, 7 of 11 genera, *Anaerotruncus*, *Eubacterium_Xylanophilum*, *Lachnospiraceae* _UCG004, *Lachnoclostridium*, *Eubacterium_Siraeum*, *Clostridia*, *and Ruminococcus* were more abundant, whereas *Oscillospiraceae* and *Lachnospiraceae*_A2 were less abundant in TDAG8^−/−^ RA mice than TDAG8^+/+^ RA mice (*p* ≤ 0.05). Within the phyla *Bacteroidetes*, the genera *Paraprevotella* and *Rikenellaceae* were more abundant in TDAG8^−/−^ RA mice than TDAG8^+/+^ RA mice (*p* ≤ 0.05) (Table 4).

For RA mice treated with CCL-2d to suppress TDAG8 expression and function [19], we detected 39 differentially abundant taxa in cecal mucus samples in healthy controls, RA mice, and RA mice with CCL-2d treatment (Figure 6B). Within the phylum *Firmicutes*, 5 of 23 genera, *Blautia*, *Marvinbryantia*, *Mycoplasma*, *Oscillibacter*, and *Tyzzerella*, were more abundant (*p* ≤ 0.05) in RA mice than healthy controls or RA mice with CCL-2d treatment. Within the phylum *Deferribacteres*, the genus *Mucispirillum* was more abundant (*p* ≤ 0.01) in RA mice than healthy controls or RA mice with CCL-2d treatment. Seven genera, *Eubacterium_Brachy*, *Eubacterium_Siraeum*, *Alloprevotella*, *Cupriavidus*, *Pseudomonas*, *Mitochondria*, and *Treponema*, were more abundant (*p* ≤ 0.05) and the genus *Anaerovoracaceae*_UCG001 was less abundant (*p* ≤ 0.05) in healthy controls than RA mice with or without CCL-2d treatment. Thus, the CCL-2d treatment restored a part of the altered gut microbial ecosystem, including a decreased relative abundance of bacteria *Eubacterium_Xylanophilum*, *Lachnospiraceae*_A2, *Anaeroplasma*, *Blautia*, *Marvinbryantia*, *Clostridia*, *Ruminococcus*, *Paraprevotella*, *Rikenellaceae*, and *Mucispirillum*, and an increased abundance of microorganisms *Eubacterium_Siraeum* and *Muribaculaceae* (Table 4).

## 3. Discussion

Alterations in the fecal microbiome between RA and healthy individuals have been reported on since the beginning of this century [3,4,5,10]. However, details of the microbiome in the colon content and mucus remained unclear for RA patients. In this study, we used samples from feces, cecal content, and cecal mucus in an RA mouse model and healthy controls to investigate the microbial composition. We generated a total of 3,461,121 sequences representing 1110 unique OTUs with a 99% Good’s coverage for all samples. Rarefaction curves showed that the sequencing depth was sufficient for further study because the samples reached the plateau phase (Figure 1A). From the rarefaction results, a minimum of 31,794 sequences per sample was used for standardizing the microbial estimations. According to the α- and β-diversity indices for the microbiome, the fecal and cecal content did not significantly differ in both healthy controls and RA mice. However, the bacterial composition in cecal mucus was significantly different from feces in all conditions. In previous studies, the mucus microbiome was also found different from that in feces from mice, humans, and *Rhesus macaque* [24,25,26]. The microbial composition in the intestine is partially correlated with that in feces, but the fecal microbiome does not represent the complete picture in the intestine [24,25,26]. In intestinal dysbiosis particularly, the represented mucus microbiome plays an important role because of a close interaction with epithelial cells and the mucus immune system [24,25,26]. In most studies, diversity indices are reduced in terms of phylogenetic diversity, species richness, and evenness in RA mice as compared to healthy individuals [9,10,27,28]. Our results agreed with the published results. The apparent decrease in microbial diversity is an important marker that indicates the association between the etiology of RA and the microbiome.

The investigation of the presence of a common core bacterial community revealed 43% of the genera in all samples. In addition, on comparing RA and healthy control samples in the different intestinal sites, the core bacterial microbiome was stable, which was more than 62% of the genera. The altered gut microbiome acts as an adjuvant criterion for clinical diagnosis to identify patients with autoimmune diseases [29,30,31,32]. In this study, at the phylum level in fecal samples, RA mice showed a decrease in *Spirochaetes* and *Palescibacteria* content and an increase in *Proteobacteria* and *Desulfobacterota* content as compared to healthy controls. However, at the phylum level in cecal mucus samples, RA mice showed a decrease in *Parabasalia*, *Bacteroidetes*, and *Spirochaetes* content and an increase in *Deferribacteres* and *Campilobacterota* content as compared to healthy controls (Table 2).

The *F/B* ratio can be used as an important indicator of the gut microbiome state and host health [28,33]. *Bacteroidetes* found in the gut mainly functions in polysaccharide metabolism and calorie absorption, whereas *Firmicutes* is important for the production of short-chain fatty acids [34]. Scher et al. found *Bacteroidetes* absent in patients with new-onset RA as compared to healthy controls [7]. The analysis of the fecal microbiome composition revealed a higher *F/B* ratio in RA than osteoarthritis patients [28]. The collagen-induced arthritis mouse model used to study the immune-priming phase of arthritis revealed a decrease in *Bacteroidetes* and an increase in *Firmicutes* content [33]. In agreement with previous studies [7,28,33], we found a higher *F/B* ratio in cecal mucus from RA than healthy control mice (Figure 5A). However, fecal samples did not show a similar trend. Thus, the *F/B* ratio could be a good indicator for mouse mucosal samples but may not apply to mouse fecal samples.

In both feces and cecal mucus, *Eubacterium_Ventriosum*, *Alloprevotella*, *Rikenella*, and *Treponema* were significantly less abundant in the RA mouse than healthy control microbiome (Figure 5B,C). Our results agreed with results from some previous studies [10,35]. Sun et al. investigated samples from 66 Chinese patients with RA and 60 healthy controls by using the bacterial 16S rDNA gene; *Alloprevotella* and *Rikenella* were less abundant in the RA than control group. *Alloprevotella* and *Treponema* were reported to produce significant amounts of short-chain fatty acids, and their abundance is negatively correlated with metabolic syndrome [35,36]. The *Alloprevotella* content was found to be positively correlated with inflammation biomarkers and the rheumatoid factor [10]. Severijnen et al. investigated arthritis-inducing properties of *Eubacterium* species and revealed a diversity in such properties among different species of the anaerobic genus *Eubacterium* in inducing joint inflammation [37]. However, the exact effect of *Treponema* and *Eubacterium_Ventriosum* on RA is difficult to determine, because the isolation and in vitro cultivation of these strains are challenging. Given that we observed a reduced abundance of *Eubacterium_Ventriosum*, *Alloprevotella*, *Rikenella*, and *Treponema* in both RA fecal and mucosal samples, these four genera could serve as microbial markers for RA progression.

In our previous study, TDAG8 gene deficiency relieved RA disease severity and chronic pain [15]. A salicylanilide derivative compound, CCL-2d, which inhibits TDAG8 function and expression, also provided similar results as TDAG8 deficiency in mice [15]. To investigate whether the deficiency of the TDAG8 gene affects the composition of the microbiome in the molecular mechanism, the inhibition of TDAG8 expression and function by gene deletion or an inhibitor was performed. The results revealed that mice with TDAG8 gene deficiency showed a restoration of the gut microbial ecosystem by significantly reducing *Eubacterium_Xylanophilum*, *Clostridia*, *Ruminococcus*, *Paraprevotella*, and *Rikenellaceae* (Table 4). A reduction in *Clostridia* was observed in RA patients using Etanercept or Sulfasalazine, drugs used to treat RA [38,39]. In this study, mice receiving TDAG8 deficiency had a decreased number of *Clostridia*. It suggested that the TDAG8 treatment of RA could be responsible for the reduction in bacterial numbers and could be potentially beneficial to RA. In previous findings, the *Ruminococcus* content was found to be correlated with intestinal inflammation and a variety of other inflammatory diseases. The inflammatory glucorhamnan polysaccharide was mainly found in *Ruminococcus* [40,41]. Additionally, RA patients showed an increased content of the genus *Rikenellaceae* [42]. The results indicated that *Clostridia*, *Ruminococcus*, and *Rikenellaceae* could be proinflammation-related microorganisms promoting RA disease progression. TDAG8 deficiency was demonstrated to reduce the number of satellite glial cells and proinflammatory macrophages that could be the cause of the change in the microbiome. Thus, TDAG8-deficient RA mice showing a reduced disease severity and RA pain could be due to the modulation of gut microorganisms affecting the pathogenesis of RA.

RA patients have chronic inflammation and persistent pain hypersensitivity to mechanical and thermal stimuli [43]. However, it is not easy to establish an animal model which reproduces all RA clinical features. In current RA models, some only have short-term inflammation, some only show unilateral hypersensitivity, some models have persistent mechanical hypersensitivity but short-term thermal hypersensitivity, and some models are not suitable in mice. Our model was adopted and modified from the model established by Gauldie et al. in 2004. Our RA mice displayed long-term inflammation and long-term bilateral pain hypersensitivity to mechanical and thermal stimuli [15,44]. RA clinical features were also found in our RA mice, such as a high concentration of [H^+^] in synovial fluid, a continuous serum IL-6 production, and an increased synovial macrophage CD68+ number that marked the disease in the chronic inflammatory state. In addition, we successfully established an RA model in both ICR and B6 mice [15,19,20]. Thus, our RA model could reproduce some of the possible mechanisms at play in RA, rather than OA or other arthritis. Our RA model started from the initiation of autoimmunity and lacked the stage of no symptoms or signs of autoimmunity. It had some limitations in the studies of some risk factors.

## 4. Materials and Methods

### 4.1. Agents

Complete Freund’s adjuvant was from Sigma-Aldrich (Darmstadt, Germany). A salicylanilide derivative compound, CCL-2d (3-(4-Chloro-2-fluorophenyl)-7-methoxy-2H-benzo[e] [1,3]-oxazine-2,4 (3H)-dione), was synthesized as described [45]. All reagents or compounds were first solved in dimethylsulfoxide, then diluted in saline before injection in animal experiments.

### 4.2. Animals

Eight to twelve-week-old ICR mice, purchased from BioLASCO Taiwan (Taipei), were housed 3–4 per cage with food and water ad libitum in a temperature- and humidity-controlled environment under a 12 h light/dark cycle (lights on at 7:00 a.m.) at National Yang-Ming Chiao-Tung University, Taiwan. TDAG8^−/−^ and TDAG8^+/+^ mice on a B6 background were generated as described [19]. The genotyping primer sequences for TDAG8^−/−^ were 5′-GAA CCA TTA GTT TGG CTC ATG TGA CTG/5′-CTT GTG TCA TGC ACA AAG TAG ATG TCC and for TDAG8^+/+^, 5′-CGA ACT CTA GCT GGC TTT TAT CCA ATA AT/5′-GAA CCA TTA GTT TGG CTC ATG TGA CTG. The experimental procedures were approved by the local animal use committee (IACUC, National Yang-Ming Chiao-Tung University, Taiwan). All animal care followed the Guide for the Use of Laboratory Animals (US National Research Council).

### 4.3. Arthritis Induction and Drug Treatment

The RA mouse model was induced as described [15]. Briefly, TDAG8^−/−^ or wild-type ICR mice were injected with 5 μg CFA in the right ankle joint once a week for 4 weeks. For CCL-2d-treated mice, CCL-2d (360 μg/kg) was administered orally (with an oral feeding needle, ST-F173 ψ0.9 × L 70 mm) weekly for 9 consecutive weeks after CFA injection. In this study, 29 mice were used; 6/29 were used as healthy controls and 3/29 were used for CCL-2d treatment; 3/29 were the TDAG8^−/−^ mouse model.

### 4.4. Sample Collection

Fecal samples were collected by using forceps and immediately frozen. The cecum was resected and opened longitudinally. The cecal contents were gently collected by using forceps without scraping the mucus surface. The outer mucus was sucked up by using a peristaltic pump with a head-cut 200 μL tip and transferred to 1 mL 0.5× phosphate-buffered saline. The mucus was immediately frozen. All samples were stored at −80 °C.

### 4.5. DNA Extraction and Sequencing

Due to the different quantity of extracted DNA from feces and mucosa and the comparison results [46], two DNA extraction kits were used. Microbial DNA from feces or cecal content was extracted by using the QIAamp PowerFecal DNA kit (Qiagen, Hilden, Germany). The extraction of DNA from cecal mucus was performed using MasterPure DNA Purification Kit (Epicentre, Madison, USA). The extracted DNA was analyzed by Health GeneTech Corp. (Taipei) for 16S rDNA gene amplification. The PCR primer set for bacterial 16S rDNA, F515 (5′-GTG CCA GCM GCC GCG GTA A), and R806 (5′-GGA CTA CHV GGG TWT CTA AT) was used to amplify the V4 region [47]. PCR amplification, library construction, and sequencing methods were described previously [48].

### 4.6. Bioinformatics Analysis

The QIIME2-2019.10 platform was used for microbial analysis [49]. Raw 16S rDNA gene sequences were demultiplexed by using the q2-demux pipeline. The sequences were then denoised and PhiX reads and chimeric sequences were filtered with DADA2 (via q2-dada2) [50]. Single-end sequences were merged by using the DADA2 plugin. Sample metadata containing information such as mouse type, treatment, and various clinical parameters for categorical and numerical formatting were used. For trimming and truncating, the DADA2 plugin was used to remove low-quality regions of sequences; the filter parameters were 19 and 214 for left forward read (R1) and 20 and 156 for right forward read (R2). To create a feature table, two plugins were used: feature-table summarize and feature-table tabulate-seqs in QIIME2. To construct a phylogeny, all amplicon sequence variants were aligned by using mafft (via q2-alignment) [51]. Alpha-diversity metrics, including observed features and Faith’s Phylogenetic Diversity, were calculated by using q2-diversity. Sequences were clustered by using the VSEARCH plugin (q2-vsearch) into operational taxonomical units (OTUs) for each sample, with a 99% sequence similarity cutoff value [52]. A summary of all taxonomic information was generated by using the q2-feature-classifier classify-sklearn naive Bayes taxonomy classifier against the Silva dataset v138 [53,54]. To standardize results, the equivalent number of sequence reads (based on the lowest number of sequences obtained from a single sample) per sample chosen by rarefaction was used for all subsequent comparisons. To determine the core microbiome, genus abundance > 0.1% was used for analysis. Venn diagrams were constructed by using Venny 2.1. Both matrices for the complete and resampled datasets were calculated and compared by applying the Mantel tests implemented in the R v3.6.3 package Vegan. For beta-diversity analysis, we determined the microbial composition diversity between individuals by using weighted UniFrac, unweighted UniFrac, Jaccard, and Bray–Curtis distance in the q2-diversity plugin [55,56]. The linear Principal Component Analysis (PCA) model was also created by using the q2-diversity plugin. Significant differences in beta-diversity were determined with QIIME by PERMANOVA, and PERMDISP was used to check for significant differences in dispersion. For featured taxa selection, we used LEfSe and Calypso [57] to calculate the linear discriminant analysis effect size (LEfSe) and random forest prediction. An LDA score of >3.0 and Kruskal–Wallis α-value of 0.05 were set as thresholds; *p* < 0.05 was considered statistically significant.

## 5. Conclusions

In this study, we compared the microbiome composition in feces and mucus samples of complete Freund’s adjuvant-induced arthritis mouse models. Four core bacterial genera, *Eubacterium_Ventriosum*, *Alloprevotella*, *Rikenella*, and *Treponema*, could be biomarkers of an altered RA microbiome in both fecal and mucosal samples. TDAG8 deficiency decreased the abundance of proinflammation-related *Eubacterium_Xylanophilum*, *Clostridia*, *Ruminococcus*, *Paraprevotella*, and *Rikenellaceae*, which reduced local mucosal inflammation to relative RA disease severity and pain. The pharmacological block of TDAG8 function by a salicylanilide derivative partly restored the RA microbiome to a healthy microbiome composition. Understanding the bacterial interaction with the host mucus in the gut and TDAG8 modulation in specific microbiota could facilitate the development of novel microbiota-based therapy.

## Figures and Tables

**Table 1:** Alpha-biodiversity for the complete datasets and the resampled datasets based on an equal number of 31,794 sequences per sample (the lowest one corresponding to CT5-Feces).

Sample	Body Site	RA	Time ^a^	Sequences ^b^	Complete Datasets				Resampled Datasets			
					N	Faith_pd	Shannon	Pielou_e	N	Faith_pd	Shannon	Pielou_e
CT1-CC	Cecum Content	No	0	74,635	253	15.17	6.76	0.85	252	15.16	6.76	0.85
CT2-CC	Cecum Content	No	0	40,683	213	13.24	6.83	0.88	213	13.24	6.83	0.88
CT3-CC	Cecum Content	No	0	44,222	230	15.47	6.82	0.87	230	15.47	6.82	0.87
CT4-CC	Cecum Content	No	12	32,782	213	17.99	6.75	0.87	213	17.99	6.75	0.87
CT5-CC	Cecum Content	No	12	36,207	211	17.82	6.64	0.86	211	17.82	6.64	0.86
CT6-CC	Cecum Content	No	12	40,825	230	14.54	6.77	0.86	230	14.54	6.77	0.86
CT1-CM	Cecum Mucosa	No	0	89,676	335	18.95	7.00	0.84	331	18.68	7.00	0.84
CT2-CM	Cecum Mucosa	No	0	86,444	312	17.83	6.85	0.83	311	17.82	6.85	0.83
CT3-CM	Cecum Mucosa	No	0	48,210	237	16.39	6.98	0.88	236	16.34	6.97	0.88
CT4-CM	Cecum Mucosa	No	0	45,748	218	16.04	6.86	0.88	218	16.04	6.86	0.88
CT5-CM	Cecum Mucosa	No	0	44,071	246	18.34	6.93	0.87	246	18.34	6.93	0.87
CT6-CM	Cecum Mucosa	No	0	46,717	236	15.26	6.88	0.87	236	15.26	6.89	0.87
CT7-CM	Cecum Mucosa	No	12	34,821	223	17.59	6.87	0.88	223	17.59	6.88	0.88
CT8-CM	Cecum Mucosa	No	12	36,539	215	16.79	6.70	0.86	215	16.79	6.70	0.87
CT9-CM	Cecum Mucosa	No	12	54,317	335	22.06	7.40	0.88	335	22.06	7.39	0.88
CT10-CM	Cecum Mucosa	No	12	40,313	250	17.64	6.74	0.85	250	17.64	6.74	0.85
CT11-CM	Cecum Mucosa	No	12	43,223	236	15.62	6.87	0.87	236	15.62	6.87	0.87
CT1-Feces	Feces	No	0	84,733	280	16.32	6.87	0.85	276	16.26	6.86	0.85
CT2-Feces	Feces	No	0	33,060	167	13.06	6.31	0.85	167	13.06	6.31	0.85
CT3-Feces	Feces	No	0	36,225	203	14.08	6.72	0.88	203	14.08	6.72	0.88
CT4-Feces	Feces	No	0	56,866	178	13.77	5.67	0.76	178	13.77	5.67	0.76
CT5-Feces	Feces	No	12	32,045	143	12.15	6.13	0.86	143	12.15	6.13	0.86
CT6-Feces	Feces	No	12	36,843	203	16.31	6.59	0.86	203	16.31	6.60	0.86
CT7-Feces	Feces	No	12	41,521	206	15.42	6.58	0.86	206	15.42	6.58	0.86
RA1-CC	Cecum Content	Yes	4	38,237	128	10.95	5.87	0.84	128	10.95	5.87	0.84
RA2-CC	Cecum Content	Yes	8	76,017	251	14.36	6.86	0.86	250	14.36	6.86	0.86
RA3-CC	Cecum Content	Yes	12	34,001	124	11.42	5.88	0.85	124	11.42	5.88	0.85
RA1-CM	Cecum Mucosa	Yes	4	87,628	283	17.09	6.65	0.82	281	17.09	6.64	0.82
RA2-CM	Cecum Mucosa	Yes	4	78,404	229	15.35	6.52	0.83	229	15.35	6.51	0.83
RA3-CM	Cecum Mucosa	Yes	8	105,853	360	19.19	7.27	0.86	359	19.19	7.26	0.86
RA4-CM	Cecum Mucosa	Yes	8	102,650	352	19.60	7.26	0.86	351	19.60	7.26	0.86
RA5-CM	Cecum Mucosa	Yes	12	93,783	279	17.09	6.39	0.79	276	17.08	6.37	0.79
RA6-CM	Cecum Mucosa	Yes	12	90,629	299	17.24	7.04	0.86	297	17.23	7.03	0.86
RA7-CM	Cecum Mucosa	Yes	12	99,632	336	16.82	7.33	0.87	335	16.81	7.31	0.87
RA8-CM	Cecum Mucosa	Yes	12	84,482	297	16.11	7.04	0.86	297	16.11	7.04	0.86
RA9-CM	Cecum Mucosa	Yes	12	79,893	296	15.76	6.96	0.85	292	15.64	6.95	0.85
RA10-CM	Cecum Mucosa	Yes	12	79,591	295	16.50	7.08	0.86	294	16.47	7.08	0.86
RA11-CM	Cecum Mucosa	Yes	12	69,997	181	12.52	6.36	0.85	181	12.52	6.36	0.85
RA12-CM	Cecum Mucosa	Yes	12	69,816	187	13.41	6.29	0.83	187	13.41	6.30	0.83
RA1-Feces	Feces	Yes	1	76,432	170	13.83	5.99	0.81	170	13.83	5.98	0.81
RA2-Feces	Feces	Yes	1	57,333	170	13.15	5.78	0.78	170	13.15	5.77	0.78
RA3-Feces	Feces	Yes	2	69,420	190	14.01	6.43	0.85	190	14.01	6.42	0.85
RA4-Feces	Feces	Yes	3	53,839	131	11.52	5.90	0.84	130	11.29	5.90	0.84
RA5-Feces	Feces	Yes	4	64,200	162	12.78	5.82	0.79	162	12.78	5.83	0.79
RA6-Feces	Feces	Yes	4	45,970	100	10.28	5.51	0.83	100	10.28	5.50	0.83
RA7-Feces	Feces	Yes	4	54,931	169	12.50	5.77	0.78	169	12.50	5.78	0.78
RA8-Feces	Feces	Yes	5	53,077	95	10.07	5.39	0.82	95	10.07	5.39	0.82
RA9-Feces	Feces	Yes	6	57,492	160	12.81	5.98	0.82	160	12.81	5.98	0.82
RA10-Feces	Feces	Yes	7	55,934	120	12.36	5.79	0.84	120	12.36	5.78	0.84
RA11-Feces	Feces	Yes	8	69,791	226	14.58	6.61	0.85	226	14.58	6.62	0.85
RA12-Feces	Feces	Yes	8	52,320	115	10.93	5.74	0.84	115	10.93	5.73	0.84
RA13-Feces	Feces	Yes	8	57,355	177	13.04	5.95	0.80	177	13.04	5.93	0.79
RA14-Feces	Feces	Yes	9	56,109	123	11.87	5.57	0.80	123	11.87	5.56	0.80
RA15-Feces	Feces	Yes	10	47,292	123	11.06	5.84	0.84	123	11.06	5.84	0.84
RA16-Feces	Feces	Yes	11	44,693	106	11.32	5.55	0.83	106	11.32	5.54	0.82
RA17-Feces	Feces	Yes	12	87,829	266	15.15	7.01	0.87	265	15.15	7.00	0.87
RA18-Feces	Feces	Yes	12	59,657	222	14.21	6.73	0.86	222	14.21	6.74	0.86
RA19-Feces	Feces	Yes	12	46,108	119	10.88	5.54	0.80	119	10.88	5.53	0.80

N is the number of operational taxonomical units. CT, RA, CC and CM indicate wild-type, rheumatoid arthritis, cecal content and cecal mucosa, respectively. Mice (8–12 weeks old) were injected with 5 µL of 100% CFA (5 µg) in the right ankle joint (ipsilateral joint) four times at 1-week intervals, followed by mechanical or thermal behavioral tests. ^a^ Weeks since experiment start. ^b^ Number of sequences.

**Table 2:** Differentially enriched taxa in mice with rheumatoid arthritis and healthy controls.

Taxa (Phylum)	*p* Value	FDR	Bonferroni	AUC	95% CI	OR No/Yes	95% CI	Delta	Fold-Change in Expression	Mean CT	Mean RA
Fecal sample											
*Spirochaetes*	0.00013	0.0016	0.0016	0.83	0.63–1	38	3.65–1005.62	2.15	159,762.9	1.12	0
*Patescibacteria*	0.0013	0.0075	0.014	0.95	0.84–1	20	2.39–447.71	1.93	2.56	1.91	0.75
*Proteobacteria*	0.0074	0.03	0.074	0.87	0.73–1	0.05	0–0.42	1.41	−2.22	0.7	1.56
*Desulfobacterota*	0.038	0.12	0.35	0.79	0.6–0.97	0.09	0–0.68	1.23	−1.59	1.28	2.04
Mucus sample											
*Parabasalia*	0.0047	0.02	0.065	0.77	0.62–0.93	13.2	1.66–287.14	1.58	11,023.73	0.09	0
*Bacteroidetes*	0.0051	0.02	0.066	0.85	0.69–1	13.33	2.07–130.86	1.34	1.1	5.82	5.29
*Spirochaetes*	0.026	0.083	0.31	0.76	0.55–0.96	19.25	2.4–425.96	1.68	8.98	1.32	0.15
*Deferribacteres*	0.000067	0.0011	0.0011	0.95	0.87–1	0.03	0–0.28	2.16	−2.44	0.63	1.54
*Campilobacterota*	0.0028	0.02	0.042	0.87	0.71–1	0.04	0–0.31	1.72	−1.49	1.99	2.96

FDR, false discovery rate; AUC, area under the receiver operating characteristic curve; CI, confidence interval; CT, healthy control; RA, rheumatoid arthritis.

**Table 3:** Composition of the fecal microbiome at weeks 1–12.

Phylum	Genus	CT–RA	*p* Value	Genus	CT–RA	*p* Value
***Firmicutes***	*Eubacterium* *_V* *entriosum*	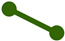	0.01	*Roseburia*	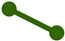	0.01
	*Lachnospiraceae*_UCG001	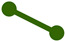	0.05	*Ruminococcus*	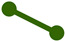	0.01
	*Monoglobus*	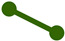	0.05	*Turicibacter*	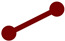	0.05
	*Oscillospiraceae*_NK4A214	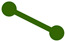	0.05	*Tuzzerella*	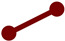	0.05
	*Bacilli*_RF39	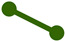	0.05			
***Bacteroidetes***	*Alistipes*	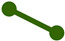	0.05	*Odoribacter*	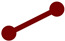	0.01
	*Alloprevotella*	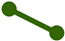	0.05	*Parabacteroides*	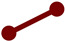	0.05
	*Rikenella*	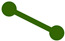	0.01	*Rikenellaceae*_RC9	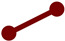	0.05
	*Muribaculum*	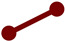	0.01			
***Proteobacteria***	*Parasutterella*	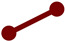	0.01			
***Spirochaetes***	*Treponema*	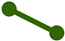	0.005	*Brachyspira*	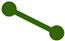	0.05
***Patescibacteria***	*Candidatus_Saccharimonas*	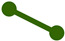	0.05	*Candidatus_Arthromis*	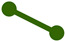	0.01

CT–RA, comparison of control and rheumatoid arthritis samples.

**Table 4:** Composition of the cecal mucus microbiome at weeks 1–12 and 12.

Phylum	Genus	Week 1–12		Week 12			
		CT–RA (ICR Mice)	*p* Value	TDAG8KO-RA (B6 Mice)	*p* value	CT–RA–CCL2d (ICR Mice)	*p* Value
***Firmicutes***	*Negativibacillus*	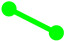	0.01	NA	NA	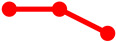	0.05
	*Eubacterium_Brachy*	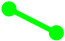	0.05	NA	NA	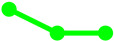	0.05
	*Eubacterium_Siraeum*	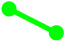	0.005	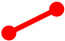	0.05	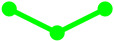	0.05
	*Eubacterium_Xylanophilum*	NA	NA	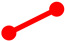	0.001	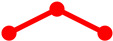	0.05
	*Intestinimonas*	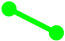	0.05	NA	NA	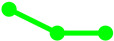	0.05
	*Lachnospiraceae*_A2	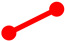	0.05	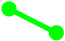	0.05	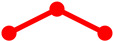	0.05
	*Anaeroplasma*	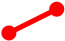	0.01	NA	NA	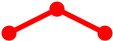	0.05
	*Blautia*	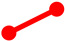	0.05	NA	NA	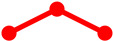	0.01
	*Lachnoclostridium*	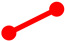	0.05	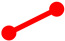	0.05	NA	NA
	*Anaerovoracaceae*_UCG001	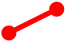	NA	NA	NA	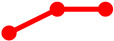	0.05
	*Marvinbryantia*	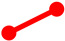	0.005	NA	NA	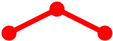	0.05
	*Clostridia*	NA	NA	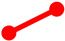	0.05	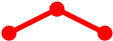	0.05
	*Oscillospiraceae*	NA	NA	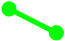	0.01	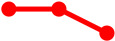	0.05
	*Ruminococcus*	NA	NA	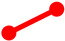	0.05	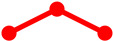	0.05
***Bacteroidetes***	*Paraprevotella*	NA	NA	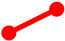	0.05	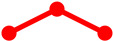	0.01
	*Alloprevotella*	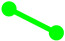	0.05	NA	NA	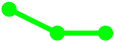	0.05
	*Muribaculaceae*	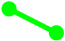	0.01	NA	NA	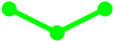	0.05
	*Rikenellaceae*	NA	NA	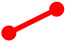	0.05	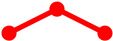	0.01
***Proteobacteria***	*Mitochondria*	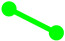	0.01	NA	NA	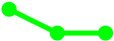	0.01
	*Pseudomonas*	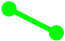	0.01	NA	NA	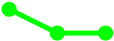	0.01
***Spirochaetes***	*Treponema*	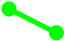	0.005	NA	NA	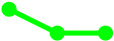	0.01
***Campilobacterot***	*Helicobacter*	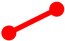	0.01	NA	NA	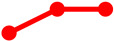	0.01
***Deferribacteres***	*Mucispirillum*	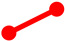	0.005	NA	NA	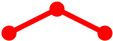	0.01

CT–RA (ICR mice), comparison of control and rheumatoid arthritis; TDAG8KO-RA (B6 mice), comparison of rheumatoid arthritis and T-cell death-associated gene 8 deficiency; CT–RA–CCL2d (ICR mice), comparison of control and rheumatoid arthritis and salicylanilide derivative.

## Data Availability

The raw sequences for this research were deposited in the Sequence Read Archive (accession no. PRJNA722190).

## Supplementary Materials

The following supporting information can be downloaded at: https://www.mdpi.com/article/10.3390/ijms23073527/s1.
