# Supplementary material for: Gut Mucosal Microbiome Is Perturbed in Rheumatoid Arthritis Mice and Partly Restored after TDAG8 Deficiency or Suppression by Salicylanilide Derivative
Source: Int J Mol Sci. 2022 Mar 24;23(7):3527. doi: 10.3390/ijms23073527 (PMC8998664; doi:10.3390/ijms23073527)
Supplement: Supplementary file 1 [file ijms-23-03527-s001.zip › ijms-1636591-supplementary.pdf]

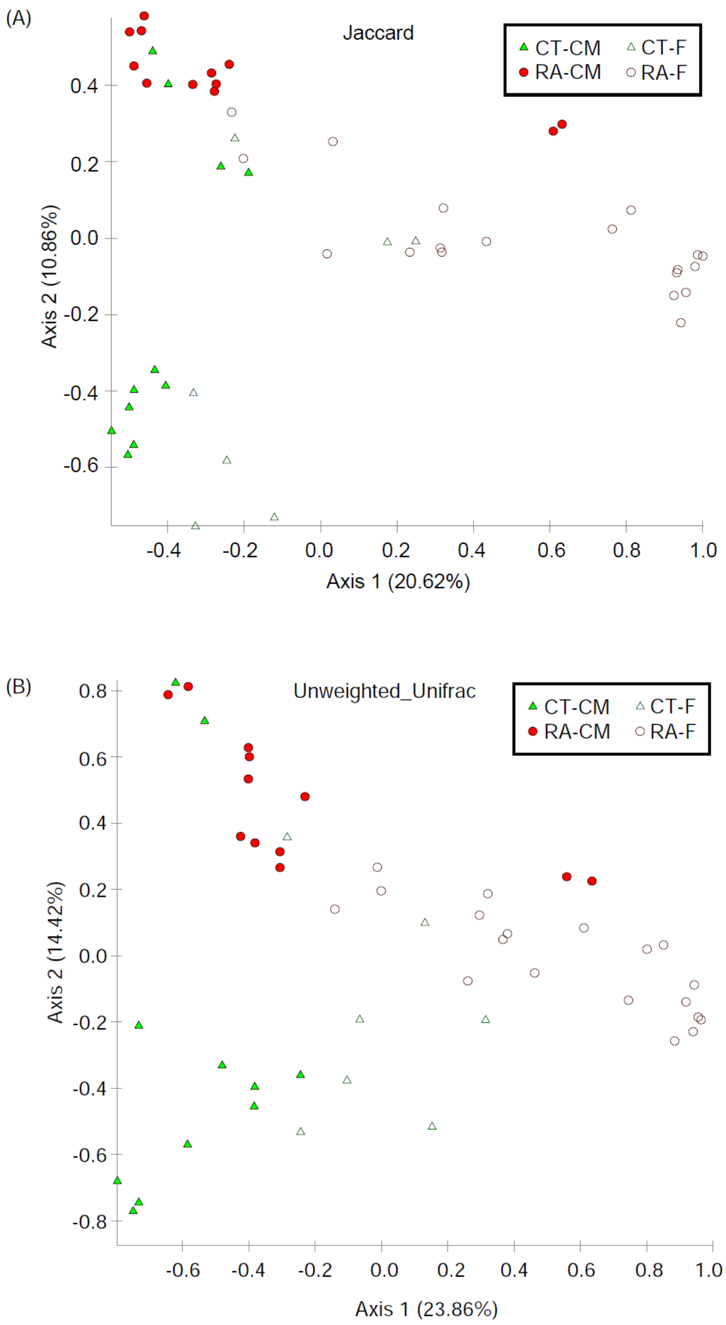

Supplemental Figure S1. *β*-diversity analysis between RA mice and healthy controls. (A) Principal coordinate analysis plot constructed by using the Jaccard distance matrix. (B) Principal coordinate analysis plot constructed by using the unweighted-UniFrac distance matrix. CT, healthy controls; RA, rheumatoid arthritis; CM, cecal mucus; F, fecal. All mice are from ICR background.

Supplemental Table S1. Pairwise-PERMANOVA and PERMDISP results based on Bray-Curtis dissimilarity for gut microbiota diversity

Bray curtis_PERMANOVA-pairwise

Group 1	Group 2	Sample size	Permutations	pseudo-F	p-value	q-value
CT-CC	CT-CM	17	999	0.32856	0.948	0.948
CT-CC	CT-Feces	13	999	1.386194	0.172	0.184286
CT-CC	RA-CC	9	999	2.57514	0.016	0.02
CT-CC	RA-CM	18	999	3.914294	0.001	0.002143
CT-CC	RA-Feces	25	999	6.10048	0.001	0.002143
CT-CM	CT-Feces	18	999	2.512023	0.009	0.012273
CT-CM	RA-CC	14	999	2.966399	0.002	0.00375
CT-CM	RA-CM	23	999	4.478852	0.001	0.002143
CT-CM	RA-Feces	30	999	10.51654	0.001	0.002143
CT-Feces	RA-CC	10	999	2.839801	0.007	0.0105
CT-Feces	RA-CM	19	999	5.325122	0.001	0.002143
CT-Feces	RA-Feces	26	999	4.429387	0.001	0.002143
RA-CC	RA-CM	15	999	1.90157	0.103	0.118846
RA-CC	RA-Feces	22	999	3.52668	0.004	0.006667
RA-CM	RA-Feces	31	999	8.655425	0.001	0.002143

Bray curtis_PERMDISP-pairwise

Group 1	Group 2	Sample size	Permutations	F-value	p-value	q-value
CT-CC	CT-CM	17	999	0.016693	0.854	0.868
CT-CC	CT-Feces	13	999	0.399979	0.496	0.744
CT-CC	RA-CC	9	999	2.980113	0.126	0.63
CT-CC	RA-CM	18	999	0.188615	0.655	0.81875
CT-CC	RA-Feces	25	999	0.03553	0.821	0.868
CT-CM	CT-Feces	18	999	0.662935	0.404	0.673333
CT-CM	RA-CC	14	999	8.777898	0.112	0.63
CT-CM	RA-CM	23	999	0.549857	0.392	0.673333
CT-CM	RA-Feces	30	999	0.016301	0.868	0.868
CT-Feces	RA-CC	10	999	4.870214	0.048	0.63
CT-Feces	RA-CM	19	999	1.2533	0.24	0.673333
CT-Feces	RA-Feces	26	999	0.311571	0.559	0.762273
RA-CC	RA-CM	15	999	1.488566	0.347	0.673333
RA-CC	RA-Feces	22	999	3.4689	0.238	0.673333
RA-CM	RA-Feces	31	999	0.663185	0.32	0.673333
